# MDM2 antagonists synergize with PI3K/mTOR inhibition in well-differentiated/dedifferentiated liposarcomas

**DOI:** 10.18632/oncotarget.16345

**Published:** 2017-03-17

**Authors:** Audrey Laroche, Vanessa Chaire, Marie-Paule Algeo, Marie Karanian, Benjamin Fourneaux, Antoine Italiano

**Affiliations:** ^1^ Université de Bordeaux, Bordeaux, France; ^2^ Institut National de la Santé et de la Recherche Medicale, Institut Bergonié, Bordeaux, France; ^3^ Department of Medical Oncology, Institut Bergonié, Bordeaux, France

**Keywords:** MDM2, CDK4, well-differentiated/dedifferentiated liposarcomas

## Abstract

**Background:**

Well-differentiated/dedifferentiated liposarcoma (WDLPS/DDLPS) are characterized by a consistent amplification of the MDM2 gene. The PI3K/AKT/mTOR pathway has been suggested to play also an important role in their tumorigenesis. Our goal was to determine whether combined MDM2 and PI3K/AKT/mTOR targeting is associated with higher anti-tumor activity than single agent alone in preclinical models of WDLPS/DDLPS.

**Methods:**

WDLPS/DDLPS cells were exposed to RG7388 (MDM2 antagonist) and BEZ235 (PI3K/mTOR dual inhibitor) after which apoptosis and signaling/survival pathway perturbations were monitored by flow cytometry and Western blot analysis. Xenograft mouse models were used to assess tumor growth and animal survival. Western blotting, histopathology, and tumor volume evolution were used for the assessment of treatment efficacy.

**Results:**

The PI3K/AKT/mTOR was upregulated in up to 81% of the human WDLPS/DDLPS samples analysed. Treatment with RG7388 and BEZ235 resulted in a greater tumor activity than either drug alone with a significant difference in terms of cell viability after 72h of treatment with RG-73888 alone, BEZ235 alone and a combination of both agents. Consistent with these observations, we found a significant increase in apoptosis with the combination *versus* the single agent treatment alone. We then analysed the in vivo antitumor activity of RG7388 and BEZ235 in a xenograft model of DDLPS. The combination regimen significantly reduced tumor growth rate in comparison with single agent alone.

**Conclusions:**

Our results represent the first in vivo evidence of synergy between MDM2 and PI3K/AKT/mTOR antagonists and represent a strong rationale to evaluate the therapeutic potential of such a combination in WDLPS/DDLPS.

## INTRODUCTION

Accounting for up to 25% of all sarcomas in adults [1], liposarcoma (LPS) is the most common soft tissue sarcoma (STS). Liposarcomas can be divided into three categories on the basis of their cytogenetic characteristics: myxoid/round cell LPS, pleomorphic LPS and well-differentiated and dedifferentiated LPS (WDLPS/DDLPS). WDLPS represent more than 40% of all diagnosed LPS and are subdivided in three main histological subtypes: adipocytic, sclerosing and inflammatory. The three main locations are the limbs (50%), the retroperitoneum (30%) and the paratesticular area (20%). Although these tumors do not metastasize, retroperitoneal and paratesticular ones are associated with a high risk of local recurrence (up to 90%) and a high a risk of dedifferentiation (up to 20%) [2, 3] whereas these risks are lower for tumors located in the limbs [4].

DDLPS are biphasic neoplasms occurring in the same age group as WDLPS, with one component being a WDLPS and the other a non lipogenic sarcoma of variable histological grade. About 90% of DDLPS arise de novo, while 10% occur in recurrence. This histological subtype is found mostly in the retroperitoneum [3, 5] and has a more aggressive behavior than WDLPS with an estimated 5-year disease-specific-survival of 44% *versus* 93% [6]. The local recurrence rate for retroperitoneal tumors can reach 80% and distant metastatic relapse is observed in up to 30% of cases [2, 7].

Surgery is the cornerstone of the primary management of WDLPS/DDLPS. Resection with R0 margin status is an achievable goal for WDLPS/DDLPS located in the limbs but is more challenging for retroperitoneal tumors for obvious anatomical reasons. Therefore, retroperitoneal tumors are associated with a higher rate of recurrence.

Systemic therapy is the most suitable approach for patients with advanced/unresectable disease. However, we have reported that the role of conventional chemotherapy such as doxorubicin, ifosfamide or trabectedin in this setting is very limited with an objective response rate of 12% and a median PFS of only 4.6 months respectively [8]). New therapeutic options are therefore urgently needed.

We and others have shown that WDLPS/DDLPS cells contain supernumerary ring or giant marker chromosomes composed of highly amplified sequences from the 12q14-15 chromosomal region [1, 9] which contain consistently the *MDM2* (12q15) and *HMGA2* (12q14.3) genes. We have also shown that the the *CDK4* gene (12q14.1) belongs to a distinct amplicon which is inconsistent but present in up to 90% cases [9].

Recently, a class of imidazoline compounds has been identified as potent and selective inhibitors of the TP53-MDM2 interaction [10]. These molecules, termed nutlins, interact specifically with the TP53-binding pocket of MDM2 and thus release TP53 from negative control. Treatment of cancer cells expressing wild type TP53 with nutlins stabilizes TP53 and activates the TP53 pathway leading to activation of TP53 target genes, cell cycle arrest, apoptosis and/or senescence. We have recently shown that the nutlins activates the TP53 pathway and decreases cell proliferation in patients with WDLPS/DDLPS [11]. However, only few patients had objective response or long-term disease stability [12].

**Figure 1:**
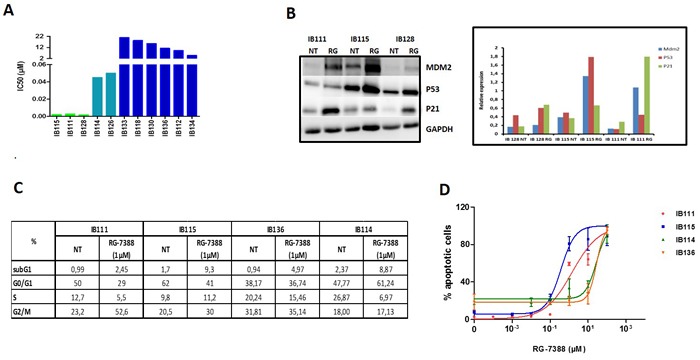
**A**. IC 50 (μM) of nutlin (RG-7388) for 11 soft tissue sarcoma cells, IB111, IB115, IB128, IB114 and IB126 are P53 wild-type, the other cell lines are P53 mutated. MDM2 is only amplified in IB115 and IB111 cells. **B**. Sensitive cells untreated or exposed to IC50 of RG-7388 (RG) were immunoblotted for MDM2, TP53 and P21 expression and quantification of the immunoblots **C**. Cell cycle profile before and after treatment with 1μM of RG7388 analyzed by PI incorporation and flow cytometry after 48h of treatment **D**. Effect of RG7388 on apoptosis in four cell lines: 2 very sensitives to RG7388 (IB111 and IB115), one with intermediate sensitivity (IB114) and one resistant cell line (IB136).

Loss of PTEN and activation of the PI3K/AKT/mTOR pathway was suggested to be involved in WDLPS/DDLPS tumorigenesis [13–14]. Interactions between the TP53 and PI3K/AKT pathways play a significant role in the determination of cell death/survival [15–19]. Indeed, the TP53-MDM2 loop is embedded in a larger network that includes AKT, a gene associated with cell survival signaling pathways. The significance of this TP53-AKT network (Supplementary Figure 1) is highlighted by the following facts: it involves two known tumor suppressor genes (TP53 and PTEN), and two oncogenes (MDM2 and AKT) [15–19]. Importantly, the TP53-AKT network is composed of two feedback loops: the mutual antagonism between TP53 and AKT which is a positive feedback loop (Figure 1), and the TP53-MDM2 negative loop (Supplementary Figure 1).

We report here a study which aims at determining whether targeting both MDM2-TP53 interaction and PI3K/AKT/mTOR pathways is associated with synergistic activity in WDLPS/DDLPS.

## RESULTS

### Aberrant PI3K/AKT/mTOR pathway activation in primary human well-differentiated and dedifferentiated liposarcomas

To test whether the PI3K/AKT/mTOR pathway is altered in human WDLPS/DDLPS, we performed immunohistochemical analysis to evaluate the expression of PTEN, a negative regulator of AKT, and of p-S6K, one crucial downstream target of mTORC1 on clinical specimens from 37 patients with well-differentiated (n=10) and dedifferentiated liposarcomas (n=27). These studies demonstrated loss of PTEN expression in 2 WDLPS (20%) and 16 DDLPS (59%) and evidence of S6 activation in 7 WDLPS (70%) and 21 DDLPS (81%) (Supplementary Figure 2). We did not observe any evidence of PTEN loss or S6 phosphorylation in lipomas (data not shown). By using comparative genomic hybridization analysis, we found that the *PTEN* gene was deleted in only 2 cases out of 24 (8%) (data not shown).

### RG7388 activates the TP53 pathway, induces significant proliferation inhibition, cell-cycle arrest and apoptosis in DDLPS cell lines

As predicted by the mechanistic model of TP53 regulation, the nutlin compound RG7388 inhibited significantly the proliferation of 5 out of 11 STS cell lines with no TP53 mutations as assessed by Sanger sequencing (IC50: 2-50 nM), but not of the 6 out of 11 cell lines with TP53 mutations (Figure 1A). The most sensitive cell lines were the DDLPS cell lines IB111, IB115 characterized by an amplification of the MDM2 gene and the extraskeletal osteosarcoma cell line IB128 with no alteration of the MDM2 gene copy numbers. In agreement with the mechanism of action of nutlins, treatment of the DDLPS cell lines with RG7388 showed an accumulation of the TP53 protein and its targets, P21 and MDM2, as revealed by Western blotting (Figure 1B) One of the main cellular functions of activated TP53 is blocking cell cycle progression in the G1 and G2 phase. Treatment of exponentially proliferating IB111 and IB115 DDLPS cell lines with RG7388 for 48 hours led to a dose-dependent cell cycle block in G2/M phase (Figure 1C). One of the other main functions of activated TP53 is induction of apoptosis. Exposure of exponentially proliferating IB111 and IB115 DDLPS cell lines to RG7388 for 72 hours led to the induction of apoptosis in a dose-dependent manner as revealed by Annexin V assay (Figure 1D). The effects on cell cycle and apoptosis were not observed in the myxofibrosarcoma IB114 and leimoyosarcoma IB136 cell lines used as controls (Figure 1C and 1D).

### NVP-BEZ235 inhibits PI3K/mTOR signaling and efficiently induces proliferation inhibition, and apoptosis in DDLPS cell lines

**Figure 2:**
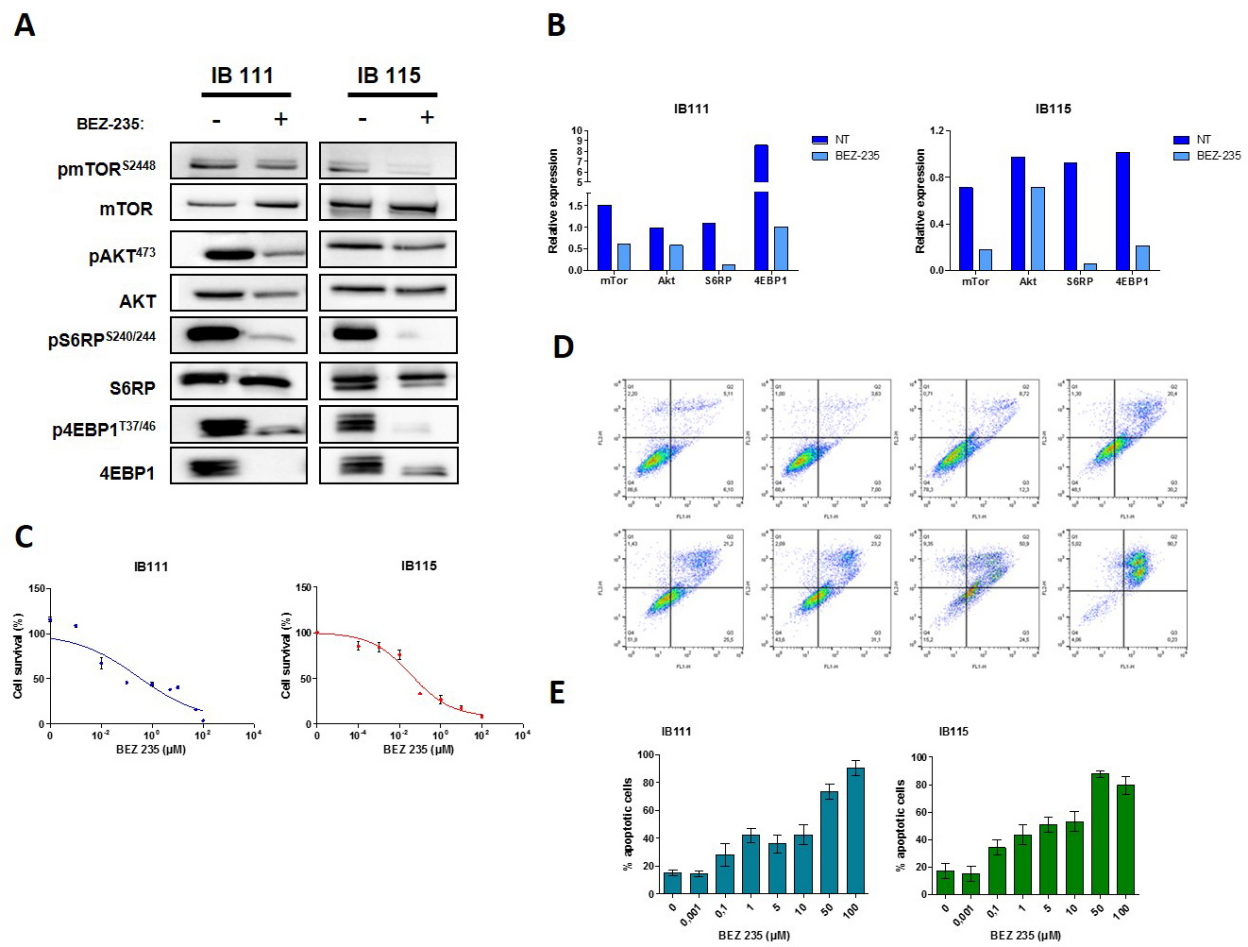
**A**. Representative blots of total and phosphorylated proteins of PI3KCA/AKT/mTOR pathway in the DDLPS cell lines untreated or exposed to BEZ-235 at 0,1μM are shown, each experiment was repeated twice **B**. Quantification of the Western blots. **C**. Effect of BEZ-235 on DDLPS cells proliferation (IB111 and IB115), the IC50 are respectively 0,3μM and 0,036μM **D**. Effect of BEZ-235 on cell death using FITC annexin-V and propidium iodide assay for IB111 cell line. **E**. quantification of apoptosis in DDLPS cells untreated or exposed at increasing doses of BEZ-235.

We confirmed that the IB115 and IB111 DDLPS cell lines exhibited high baseline levels of PI3K/AKT/mTOR signaling measured by p-AKT, p4EBP1, pMTOR and phosphoS6 (Figure 2A and 2B). Treatment of the IB115 and IB111 cells with NVP-BEZ235 was able to inhibit significantly the phosphorylation of Akt, Ser2448 mTOR, Ser240/244 S6 and 4EBP1 (Figure 2A and 2B). Correspondingly, 10^-4^ to 100 μmol/L BEZ235 decreased DDLPS cell growth in a concentration-dependent manner, (Figure 2C). Exposure of exponentially proliferating DDLPS cell lines to for 72 hours also led to the induction of apoptosis in a dose-dependent manner as revealed by Annexin V assay (Figure 2D and 2E). Cell-cycle analysis demonstrated no cell-cycle arrest at G0-G1 following 48 hours of exposure to increase doses of BEZ-235 (data not shown).

### Combined inhibition of PI3K/AKT/mTOR and MDM2 enhances growth inhibition and apoptosis of DDLPS cells

**Figure 3:**
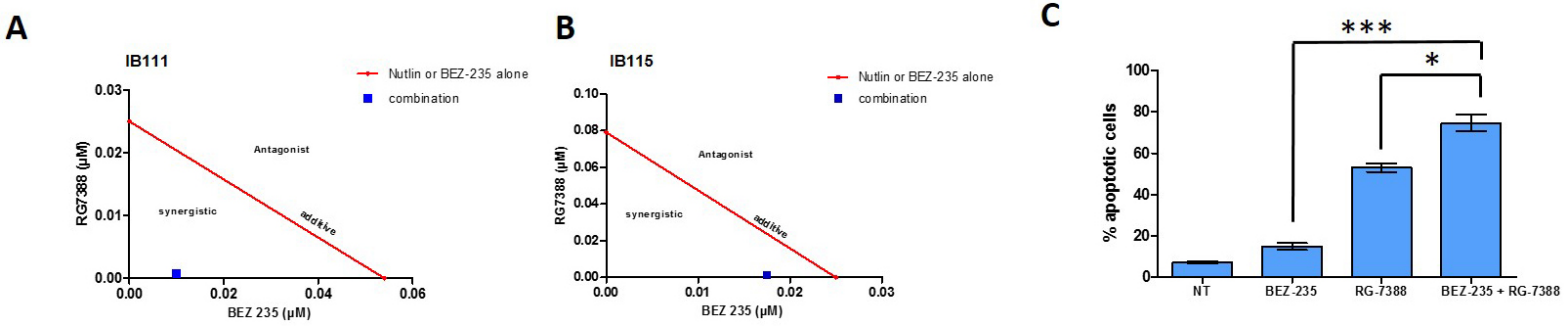
Isobologram representation for the IB111 **A**. and IB115 **B**., cell lines, the dots located lower left, on the diagonal line, or upper right indicate synergism, additivity, and antagonism, respectively, combination index was calculated and CI = 0,22 and 0,71 respectively. **C**. quantification of apoptotic cells after 72h of treatment with BEZ-235 or nutlin alone or with the two drugs in combination. Apoptosis induction was analysed using FITC anexin-V and propidium iodide assay in IB115 cells.

**Figure 4:**
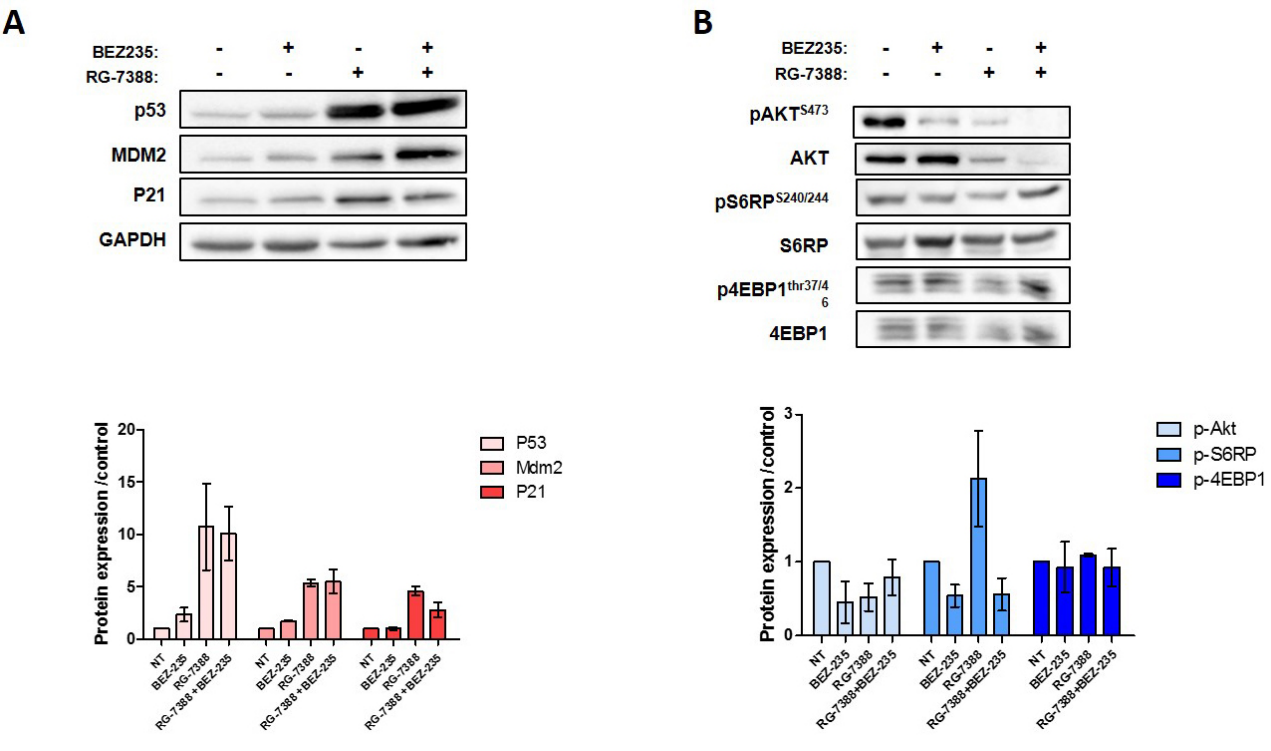
**A.** Western blot analysis of P53 proteins pathway in IB115 cell line untreated or exposed to BEZ-235 and/or RG-7388 at IC50 **B**. Western blot analysis of PI3K pathway protein phosphorylated and total in IB115 cells treated with drug alone or in combination at IC50. Each experiment was repeated 3 times.

Because the high level of PI3K/AKT/mTOR signaling can affect the anti-survival and pro-apoptotic effects of RG7388, we investigated whether inhibition of this pathway by BEZ235 would increase the response DDLPS cell lines to RG7388. As shown in Figure 3A and 3B, combination of both drugs after 72h exposure is synergistic in DDLPS cells (IB111 and IB115). Consistent with these observations, a quantitative confirmatory apoptosis assay using flow cytometry revealed that apoptosis induction was significantly higher when DDLPS cells were treated with the combination of RG7388 and BEZ235 than with single agent alone (Figure 3C). TP53 has been shown to inhibit the PI3K/AKT/mTOR pathway. To understand the mechanisms involved in the synergy between RG7388 and BEZ235 we analysed their impact on TP53 and PI3K/AKT/mTOR signalling. BEZ235 induced only a moderate increase of TP53 protein expression (Figure 4A). We found also that the PI3K/AKT/mTOR pathway remained active after treatment with RG7388 despite induction of TP53 (Figure 4B). However, the combination of both drugs induced a strong concomitant induction of TP53, P21 and inhibition of the PI3K/AKT/mTOR pathway.

### *In vivo* Activity of RG7388 and BEZ235 against tumor growth

**Figure 5:**
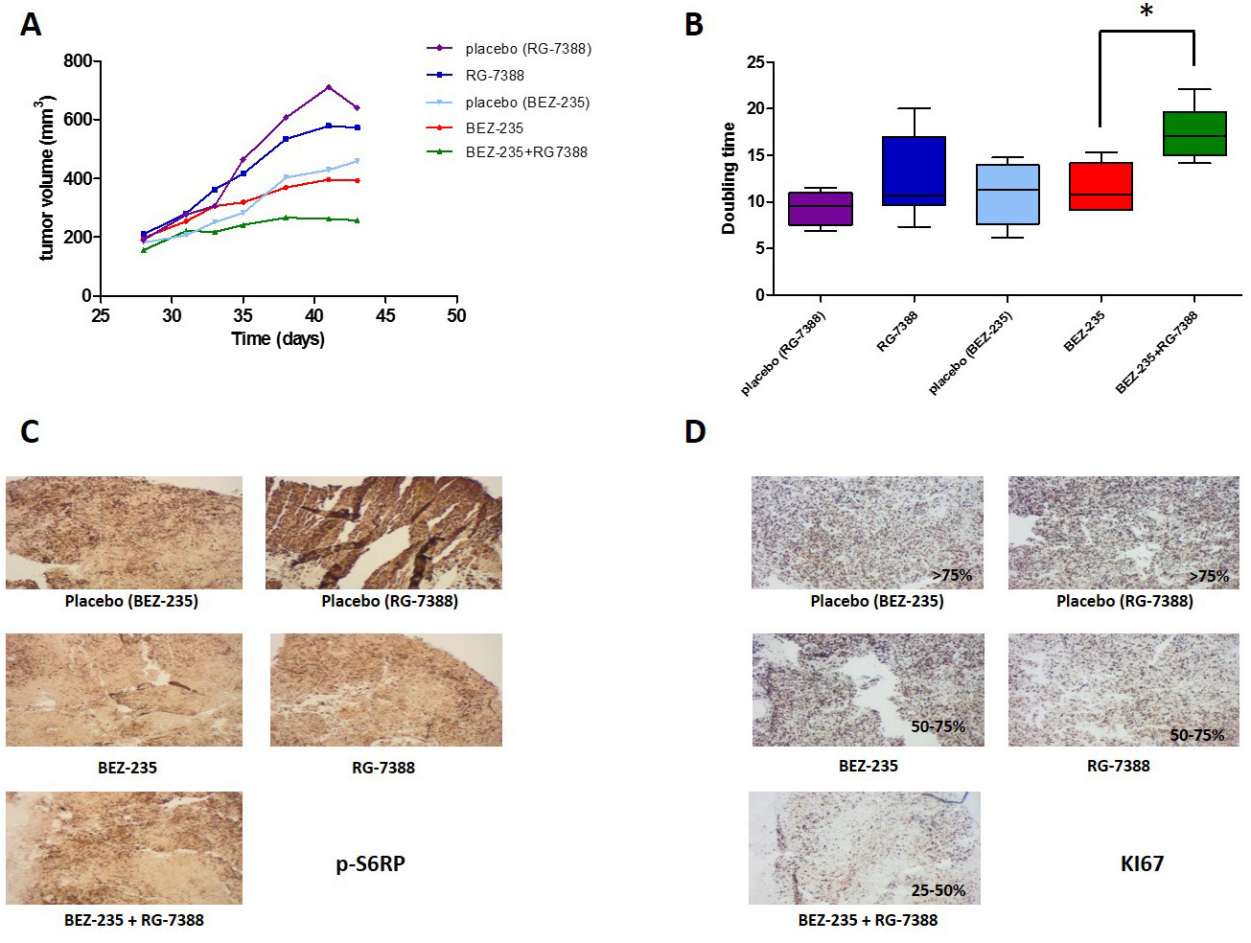
**A.** Effect of combination of RG-7388 and BEZ-235 on tumor growth, **B**. the doubling time was calculated from the tumor progression curve; **C**. immunohistochemistry on tumor tissues with p-S6RP and **D**. KI67 antibodies

The *in vivo* antitumor activity of NVP-BEZ235 and RG7388 was then analysed in a xenograft model from the IB115 cell line. When tumors were measurable and reached an average volume of 0.1 cm3, the animals were treated with RG7388 alone, NVP-BEZ235 alone and a combination of both. The combination regimen significantly reduced tumor growth rate and time to progression in comparison with single agent alone (Figure 5A and 5B). No significant changes in the weight of the tumor-bearing mice were observed after treatments with NVP-BEZ235 (data not shown). Specific effects of NVP-BEZ235 on the PI3K/Akt pathway *in vivo* were confirmed by immunohistochemistry on tumor tissues at the end of treatments by using immunohistochemical evaluation of p-S6RP^ser240/244^ in controls or in tumors derived from mice treated with the different regimens. (Figure 5C). No significant difference in staining intensity was observed when combining BEZ 235 and RG7358 *versus* BEZ235 as a single agent. (Figure 5C). Consistently with the cytostatic effects induced by NVP-BEZ235, KI-67 immunostaining was also significantly reduced following treatment with the combination *versus* single agent inhibitor (Figure 5D).

## DISCUSSION

Well-differentiated/dedifferentiated liposarcomas are the most frequent subtypes of STS and are characterized by a specific amplification of MDM2 (12q14-15) [1, 8]. Stimulating the tumour suppressor activity of wildtype TP53 protein has long been shown to eradicate tumour cells in animal models, which makes the molecule an attractive therapeutic target for drug development. Inhibition of the MDM2TP53 interaction with synthetic molecules might therefore lead to the accumulation of active TP53 followed by the death of the tumour cells from apoptosis as shown *in vitro* [10]. Nutlins represent a class of antagonists that inhibited the MDM2-TP53 complex and first described in 2004 [10]. This class of agents can disrupt the MDM2-TP53 interaction by binding a well-defined hydrophobic cleft of MDM2 which represented the binding site for TP53, liberating functional TP53 [10]. There are only limited pre-clinical data regarding the anti-tumor activity of nutlins in sarcomas [20–22]. Most of them were based on bone sarcoma cell lines and not on STS models. We have recently shown that MDM2 antagonist activates the TP53 pathway and decreases cell proliferation in patients with MDM2-amplified liposarcoma. However, in this study, 25% of patients displayed primary resistance [11].

The combination of genotoxic drugs with nongenotoxic nutlin has been reported to synergistically activate TP53 functions, paving the way for the potential use of nutlin in combinatorial drug therapy [23, 24]. Ohnstade and all investigated *in vitro* the effect of Nutlin- in combination with the current cytotoxic drugs (e.g. Doxorubicin, Cisplatin and Methotrexate) on sarcoma cells [25]. Among the drug tested, only doxorubicin is used in the management of WDLPS/DDLPS. The authors found a synergy that supported the development of this combination in the clinics. We have indeed recently reported through a phase 1 study that combination therapy with doxorubicin and the nutlin compound RG7112 resulted in an apparent potentiation of TP53 activation in an unselected population of patients with advanced STS [26]. However, due to the toxicity profile of doxorubicin, this combination resulted in a high rate of grade 3 or 4 haematological toxicity with 60% and 45% of patients experiencing severe neutropenia or thrombocytopenia respectively precluding its future development.

Therefore, combination of nutlin with targeted nongenotoxic drug may represent a more relevant approach. Several studies have reported dysregulation of the PI3K/AKT/mTOR pathway in WDLPS/DDLPS [13–14]. By using a zebrafish model, Guttierez et al showed that AKT activation and TP53 dysregulation collaborate in WDLPS/DDLPS tumorigenesis [13]. Interestingly, they observed as in our study that inhibitors of the PI3K-AKT-mTOR pathway such as BEZ235 were able to impair viability in human WDLPS and DDLPS cells. Smith et al demonstrated by using xenograft model of DDLPS that activation of the PI3K-AKT-mTOR pathway in these tumors was associated with a frequent loss of PTEN expression [14]. As in our study, CGH analysis of the patient tumors from which the xenografts were derived showed no genetic alterations at the PTEN locus, suggesting the involvement of epigenetic mechanisms. The cross talk between the PI3K/AKT/mTOR pathway, TP53 and MDM2 play an important role in the regulation of pro-apoptotic and anti-apoptotic signals. For instance, AKT mediates negative control of TP53 levels through enhancing MDM2 (murine double minute 2)-mediated targeting of TP53 for degradation [27]. Moreover, PTEN is able to regulate the function of TP53 by both phosphatase-dependent and -independent mechanisms [28]. Our data represent the first *in vivo* evidence that inhibition of PI3K/AKT/mTOR signalling can augment TP53-mediated apoptosis induced by MDM2 antagonist in human tumors. Interestingly, this results are in agreement with those of Saiki et al showing that inhibition of the PI3K pathway, including PI3K itself, AKT, or mTOR, yielded profound *in vitro* synergy in combination with MDM2 inhibition in several epithelial tumors [29]. The molecular mechanisms underlying the observed synergy between TP53-MDM2 interaction and PI3K/AKT/mTOR inhibitions remains to be elucidated. Recent studies have demonstrated that one important tumor suppressive function of TP53 is related to its ability to inhibit mTOR activity [30]. In our study, western blot analysis suggested that mTOR pathway remains active after treatment with RG7388 in WDLPS/DDLPS despite induction of TP53. We observed also that treatment with BEZ235 resulted in a moderate upregulation of TP53 expression. These findings are in agreement with those from Herzog et al indicating an upregulation of TP53 in HNSCC cell lines treated with the dual inhibitor PI3K/mTOR PF-04691502 [31]. Therefore, upregulation of the PI3K/AKT/mTOR pathway in cancer may be linked to repression of TP53 expression that can be reversed at least in part by inhibitors of the PI3K/AKT/mTOR signaling.

In conclusion, our data represent a strong rationale to evaluate the therapeutic potential of dual PI3K/mTOR inhibitors as potentiators of TP53-MDM2 interaction antagonists in WDLPS/DDLPS. Given the devastating nature of this disease, the confirmation of our data in the clinical setting would represent a strong achievement for the sarcoma community.

## MATERIALS AND METHODS

### Patients and samples

We analyzed 37 cases of WDLPS (n=10) and DDLPS (n=27) with available paraffin-embedded material. All the cases were reviewed by a pathologist expert in the field of soft-tissue tumors. Immunohistochemistry for PTEN (CST 9559; 1:100, Cell signaling), and phosphorylated ribosomal protein S6 kinase (p-S6K) (CST 5364; 1:100, Cell signaling), was performed according to each manufacturer's recommendations. PTEN expression was assessed semi-quantitatively from score 0 (absence of expression) to 1 (decreased expression in tumoral cells compared with normal endothelial cells) and 2 (same intensity of staining in tumoral and normal cells). A 20% cutoff value for the detection of positive nuclear reactivity was selected for p-S6K.

### Cell lines

All the cell lines used in this study (Supplementary Table 1) have been derived from human surgical STS specimens in the laboratory of Pr Jean-Michel Coindre and Dr Frédéric Chibon (Institut Bergonié, Bordeaux, France) after obtaining patient consent. All cell lines were cultured in RPMI 1640 medium, GlutaMAX^TM^ Supplement (Life Technologies) supplemented with 10% (v/v) fetal bovine serum (FBS), Penicillin/Streptomycin 1%, and Normocin 0.2% (InvivoGen) at 37°C with 5% CO_2_. Cells were routinely passaged every 2 or 3 days.

### Study reagents

RG7388 (MDM2-TP53 interaction inhibitor) was supplied by Roche and BEZ235 (dual PI3K/mTOR inhibitor) was purchased from Selleck Chemicals (Houston, TX, USA). Cultured cells were treated for 72 hours with medium change and fresh drug as indicated in figure legends.

### Cell viability assay

Cells were seeded in triplicate at 3000 cells/well into 96-well plates, cultured with fresh growth medium for at least 24 hours and treated with a range of increasing concentrations of drugs for 72 h. After incubation period, 2-Deoxyglucose (2-DG),3-(4,5-Dimethylthiazol-2-yl)-2,5-diphenyltetrazolium bromide (MTT, Sigma-Aldrich Chimie, Saint-Quentin-Fallavier, France ) was immediately added to the wells at a final concentration of 0.5 mg/mL and the cells were incubated for 3 h. Then, supernatant was discarded, 100 μL of dimethyl sulfoxide (DMSO, Sigma-Aldrich Chimie, Saint-Quentin-Fallavier, France was added and the absorbance was monitored using a Flexstation 3 Plate reader (Sunnyvale, CA, USA) at 570 nm with 630 nm as reference. IC_50_ was calculated with GraphPad Prism software version 5.00 for Windows (GraphPad Software, La Jolla California USA). Each experiment was repeated at least 3 times.

### Cell apoptosis assay

Cells (2×10^5^/well) were seeded in 6-well plates and treated for 72 hours with several specific drug concentrations. After treatment, cells were washed once with PBS and labeled with annexin-V-FITC and propidium iodide (PI) according to the manufacturer's protocol (BD Biosciences, San Jose, CA, USA). Then, cells were analyzed with a FACSCalibur flow cytometer (BD Biosciences, San Jose, CA, USA. The percentage of cells in early apoptosis (annexin-V positive, PI negative) and cells in late apoptosis or necrosis (annexin-V and PI positive) was calculated using FlowJo version 7.6.3 for Windows (Tree Star Inc, Ashland, OR, USA. Data are represented as mean ± SEM values, based on 3 independent experiments.

### Western blotting

Treated and control whole cells were harvested using 60 μL Radio-ImmunoPrecipitation Assay (RIPA) lysis buffer. The lysate was centrifuged (13 000 rpm, 15 min, 4°C) and the supernatant stored at -80°C for later use. Total proteins (30 μg) were electrophoresed on 8, 12 or 15% dodecyl sulfate (SDS) polyacrylamide gel and transferred onto polyvinylidene difluoride (PVDF) membranes. Blots were probed overnight at 4°C in 5% BSA in PBST (Phosphate 100 mM, KCl 27 mM, NaCl 1.37 M pH 7.4 after 1X dilution; 0.2% Tween-20) with primary antibody to p-AKT^ser473^ (1: 1000, CST 4060), AKT (1: 1000, CST 4685), p-S6RP^ser240/244^ (1: 1000, CST 2215), S6RP (1: 1000, CST 2217), 4E-BP1 (1: 1000, CST 9452), p-4E-BP1 (1: 1000, CST 9459), anti-P53 (1: 200, Santa Cruz sc-126); anti-Mdm2 (1: 500, Calbiochem IF2); anti-P21 (1: 33, Calbiochem) and glyceraldehyde-3-phosphate dehydrogenase (GAPDH, SC-51907),

Horseradish peroxidase-conjugated secondary antibody (Santa Cruz)were diluted 1:5000. Bound antibodies were visualized by Fusion Fx7 (Fisher Bioblock Scientific, Waltham, MA, USA) using Imobilon^TM^ Western (Millipore Corporation, Billerica, MA, USA), an enhanced chemiluminescence detection kit. The resulting bands were analyzed and quantified by ImageJ® 1.49g software (National Institute of Health, Bethesda, WA, USA). GAPDH served as a loading control. Each membrane was reused 2 times after desaturation in glycin buffer (6.6mol/L, pH 2) at 56°C for 30 min.

### Drug synergy assays

Cells were treated with single drugs and combination of two drugs for 72 h. To confirm synergistic effects between two drugs, a diagonal constant ratio combination design was realized according to Chou and Talalay proposition [32]. Cells were incubated with a 2-fold serial dilution with several concentration above and below of the IC_50_ of the two drugs at a constant ratio. After incubation period, MTT was immediately added to the wells and the absorbance was monitored using the Flexstation 3 Plate reader. The analysis of synergy assay was done by the isobologram and combination index (CI) methods, derived from the median-effect principle of Chou and Talalay. The combination effects of the two agents can be summarized as follows: combination index (CI) < 1 (under the curve), CI = 1 (near the curve), and CI > 1 (above the curve) indicates synergistic, additive and antagonistic effects respectively. Synergy experiments were repeated at least three times.

### Animal studies

All animal experiments were performed with the approval of the institutional animal use and care committee. IB115 cells (5 × 10^6^ cells/200μL) were inoculated subcutaneously into the right flank of Ragγ2C-/- mice (n=10). Once palpable, tumor volumes were calculated using the following formula: length x width^2^ / 2. After tumors reached approximately 200 mm^3^ in average size, animals were treated by oral gavage for the two drugs every day; briefly BEZ was dissolved in 1 volume of NMP (1-methyl-2-pyrrolidone) in a 100°C water bath, then 9 volume of PEG300 (Sigma-Aldrich, St Quentin Fallavier, France) were added. The Nutlin was dissolved in a solution supplied by Roche. Mice were randomized to receive 40mg/kg BEZ, 50 mg/kg Nutlin or both drugs in combination. Three weeks after drug administration, the mice were sacrificed and tumors were excised and harvested in 10% paraformaldehyde. Tumor progression was analyzed with GraphPad Prism software. Paraffin sections were incubated with anti-p-S6RP^ser240/244^ (CST 5364; 1:100) and anti-Ki-67 (Ventana 790-4286; 1:100) antibodies. Percentage of labelled cells was estimated by a pathologist expert in soft-tissue tumors. The intensity of p-S6RPser240/244 staining was scored as follows: 0, negative; 1+, weak staining; 2+, intermediate staining; 3+, strong staining. Scoring was performed without knowledge of tumor response. Tissue pictures was carried out with an Olympus CKX41 (x100) using image capture cellSens Entry software version 1.14 (Olympus, Rungis, France) for Windows.

## SUPPLEMENTARY MATERIALS FIGURES AND TABLES


