# Supplementary material for: MDM2 antagonists synergize with PI3K/mTOR inhibition in well-differentiated/dedifferentiated liposarcomas
Source: Oncotarget. 2017 Mar 17;8(33):53968–77. doi: 10.18632/oncotarget.16345 (PMC5589555; doi:10.18632/oncotarget.16345)
Supplement: Supplementary file 1 [file oncotarget-08-53968-s001.pdf]

MDM2 antagonists synergize with PI3K/mTOR inhibition in well-differentiated/dedifferentiated liposarcomas

Supplementary Material

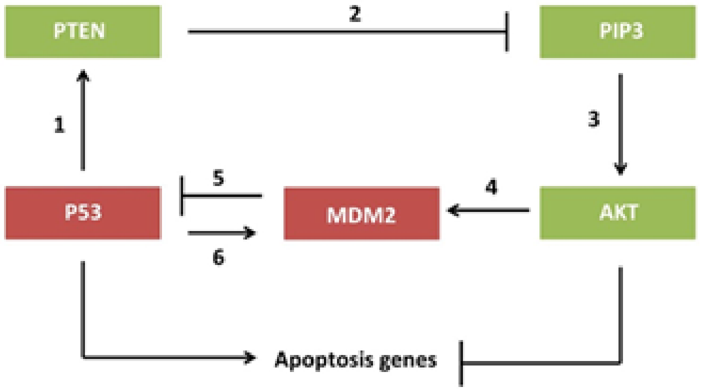

Supplementary Figure 1: Interactions between the p53 and PI3K/AKT pathways. An arrow means a pathway that leads to activation or upregulation; a hammerhead represents inhibition or downregulation. AKT is antagonized by p53 via edges 1–3, and p53 is antagonized by AKT via edges 4–5. A p53-MDM2 negative feedback loop is shown by edges 5 and 6.

Supplementary Table 1:

Cell lines	histotype	P53 statut	Mdm2	RG7388 IC50 (µM)
IB115	DDLPS	WT	amplified	0.002
IB111	DDLPS	WT	amplified	0.003
IB128	osteosarcoma	WT	normal	0.002
IB114	Myxofibrosarcoma	WT	gain	0.045
IB126	Synovial sarcoma	WT	gain	0.05
IB133	LMS	Mut (homozygous deletion exon2-3)	lost	20.8
IB118	LMS	Mut (exon 8-9, c.833 C>T p.PRO278Leu)	normal	18.1
IB130	Pleomorphic LPS	Mut (exon 8-9, c.833 C>T p.PRO278Leu)	lost	15.5
IB136	LMS	Mut (homozygous deletion)	gain	11.2
IB112	LMS	Mut (homozygous deletion)	normal	9.1
IB134	Uterine LMS	Mut (exon 5-6, c.645 T>G p.Ser215arg)	gain	4.7

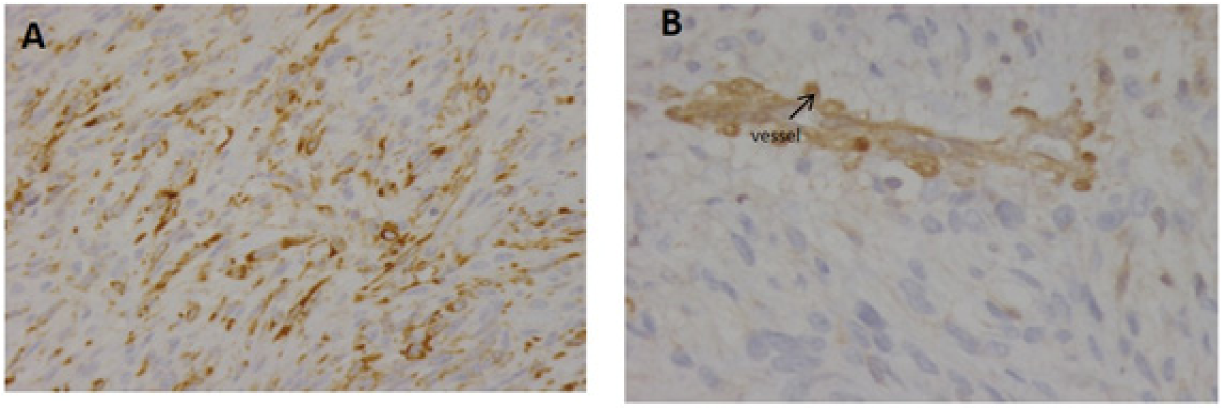

Supplementary Figure 2. Dedifferentiated liposarcoma : (A) strong intensity staining for phospho-S6RP (× 400). (B) Loss of PTEN expression by tumor cells (positive control: endothelial cells)
